# Emergency medicine board pass rates and medical residents’ experiences following the introduction of the small group learning approach in Qatar

**DOI:** 10.5116/ijme.5cb2.e936

**Published:** 2019-04-24

**Authors:** Khalid Bashir, Mohammed Talha Bashir, Shahzad Anjum, Stephen Thomas

**Affiliations:** 1 Department of Emergency medicine, Hamad General Hospital, Doha, Qatar; 2 University of Aberdeen School of Medicine and Dentistry, Scotland, UK

**Keywords:** Emergency medicine, board pass rates, medical residents’ experiences, small group learning, Qatar

## To the Editor

The traditional lecture (TL) approach has been the standard delivery method for medical education for a long time and has facilitated the training of a large number of students in a relatively short period of time.^1^ Pedagogical literature, however, criticizes the “chalk and board” approach of TL and shows that it has little benefit in enhancing students’ knowledge and skills.^2^ A further criticism is that engaging students in learning using the TL approach is not effective, and their average attention span is 10–15 minutes.^3^  However, innovative learning strategies suggest a more interactive delivery to improve knowledge transfer and student engagement.^4^  For example, although small-group learning (SGL) is recommended as an alternative to TL, the TL approach was emphasized in the emergency medicine (EM) residency training program in Qatar until 2014. In 2015,’the Board-Prep Curriculum’ for medical residents concluded that the SGL approach was superior to the TL approach. This could be due to two studies^5^^, ^^6^   that have already shown that the SGL approach has contributed to residents enhancing their communication and problem-solving skills. However, the drawbacks to the approach include its cost inefficiency, which is due to the need for the higher teacher-student ratio.^5^ In addition, no previous study has investigated the association between EM board pass rates and residents’ perspectives following the use of the SGL approach in Qatar. Because of this, we compared EM board exam scores in emergency medicine residents who were trained by SGL with those who were trained by TL. Our study was conducted at Hamad Medical Corporation, a tertiary care center in the State of Qatar that hosts a four-year EM residency program which has been accredited by the international branch of the USA-based Accreditation Council for Graduate Medical Education (ACGME-I). The data were collected over a period of six years (2012-2017). EM residents’ perspectives of both approaches (SGL and TL)  were explored using semi-structured interviews. Information on EM board pass rates was obtained from ‘the Official Arab Board of the EM Report’. The overall pass rate  for SGL (n=30) was 76.7% and for the LT group (n=33) was 75.7%. No significant differences in the exam results were reported with the two different approaches. However,  the  interviews showed that the EM residents  preferred  the SGL to TL approach, describing it as “engaging, intellectually motivating”, and also it “helps with improvement and retention of knowledge.”

Finally, in our study, a number of important limitations need to be considered.  First, the study has only considered the TL and SGL approaches and has not explored the effect of other extraneous variables on the results, such as attending exam preparation courses.  Second,  the number of EM residents (Five medical residents) who were interviewed was relatively small, and these five  EM residents  all  preferred the SGL approach to the TL approach, and  hence it is possible to argue that other residents, who did not participate in our interviews,  might have preferred other approaches including TL. If the debate is to be moved forward,  further investigation and experimentation into the SGL approach are strongly recommended.

### Conflict of Interest

The authors declare that there are no conflicts of interest.
